# A retrosynthetic analysis algorithm implementation

**DOI:** 10.1186/s13321-018-0323-6

**Published:** 2019-01-03

**Authors:** Ian A. Watson, Jibo Wang, Christos A. Nicolaou

**Affiliations:** Discovery Chemistry, Lilly Research Laboratories, Eli Lilly and Company, Indianapolis, IN 46285 USA

**Keywords:** Retrosynthetic analysis, Chemical synthesis, Synthetic route design, Reaction informatics

## Abstract

The need for synthetic route design arises frequently in discovery-oriented chemistry organizations. While traditionally finding solutions to this problem has been the domain of human experts, several computational approaches, aided by the algorithmic advances and the availability of large reaction collections, have recently been reported. Herein we present our own implementation of a retrosynthetic analysis method and demonstrate its capabilities in an attempt to identify synthetic routes for a collection of approved drugs. Our results indicate that the method, leveraging on reaction transformation rules learned from a large patent reaction dataset, can identify multiple theoretically feasible synthetic routes and, thus, support research chemist everyday efforts.

**Electronic supplementary material:**

The online version of this article (10.1186/s13321-018-0323-6) contains supplementary material, which is available to authorized users.

## Introduction

Research needs for chemical synthesis predictability, synthetic route planning and reaction optimization has motivated the development of several computational tools in recent years [3, 15, 19]. Traditionally, these tools implement methods based on precedent reaction look-up or retrosynthetic analysis solutions [8]. The former relies on the presence of a collection of reactions and attempts to match the query structure to a known reaction product (Reaxys; Scifinder). When a match is identified the original reaction is retrieved and, often, a search in available structural databases is performed to define availability of reactants. Retrosynthetic analysis (RA) approaches, first introduced through the pioneering work of Corey [5], use chemical reaction rules to deconstruct query structures into reactants followed by a search for availability in structure collections [18]. The preparation of the deconstruction rules may either be assigned to human experts or take place via computational, data-driven processes [14]. Both reaction look-up and retrosynthetic analysis approaches may iterate by using each reactant as a new query structure.

In a typical RA scenario, given a target chemical structure, the process initiates a recursive decomposition loop using available synthetic knowledge. In each step, the input chemical structure is broken into fragments which are complemented with functional groups necessary for the reaction to take place. The virtual reactants, referred to as synthons in the remainder of this manuscript, can then be matched against a database of available building blocks. The process ends when synthons match available building blocks, when the structure under investigation cannot be broken down further or when a predefined search depth has been reached.

Key components of the above process include a database of synthetic reactions to serve as the source of synthetic knowledge, and, a collection of available building blocks which serves as a look up for synthons. The proliferation of chemical structure databases renders the latter component easy to address. For example, researchers can nowadays freely access building block collections advertised as “readily available” from numerous chemical structure vendors [7]. In addition, researchers associated with the pharmaceutical industry or sizeable academic institutions often have access to inhouse chemical sample management databases of significant size. Access to a comprehensive, free, reliable source of chemical reactions has been more challenging since such data has been typically described in textual form in laboratory notebooks, journal publications and patents. Collections prepared by large publishing houses and professional organizations are only provided on a fee-for-service basis while laboratory notebooks are often difficult to search since their primary design purpose has been recording experiments while they are executed, not comprehensively searching a posteriori. Of immediate practical use is work reported by Lowe et al. [9] to extract reactions from patent applications to the United States Patent and Trade Office (USPTO) and make these reactions available in the public domain [10].

In this paper we describe our efforts to develop a data-driven retrosynthetic analysis engine aiming to provide synthetic routes for input chemical structures. We thoroughly discuss all implementation aspects including design decisions and algorithmic details and present results from the training of this engine and the application to a collection of approved small molecule drugs. This tool, originally developed to serve inhouse needs and currently in operation, is provided to the community in an effort to facilitate research in synthetic route design and reaction informatics in general. Emphasis has been placed on developing a tool agnostic to reaction and reactant data specifics so that interested parties can train and apply the method to any reaction source and building block collection appropriately formatted.

## Conceptual design

The Retrosynthetic Analysis (RTSA) method is a computational, data-driven approach designed to identify potential synthetic routes for a structure of interest, referred to in this manuscript as the hypothesis structure. The method uses as input a dataset of reaction examples to prepare retrosynthetic analysis rules used to decompose hypothesis structures into virtual building blocks. Similar to all data-driven methods, the performance of RTSA depends heavily on the supplied input data quality. For the purposes of the work described in this paper we use the publicly available USPTO reaction set which can be readily accessed and used [10].

The operation of RTSA has two distinct phases. The training phase, RTSA-Train, consumes all available reaction data and produces a collection of chemical structure decomposition rules and information required for synthetic route design. RTSA-Design, receives the hypothesis structure(s) and designs possible synthetic routes using the collection of decomposition rules obtained during training. In the following sections the two phases of RTSA are described in detail.

### RTSA-train

**Fig. 1:**
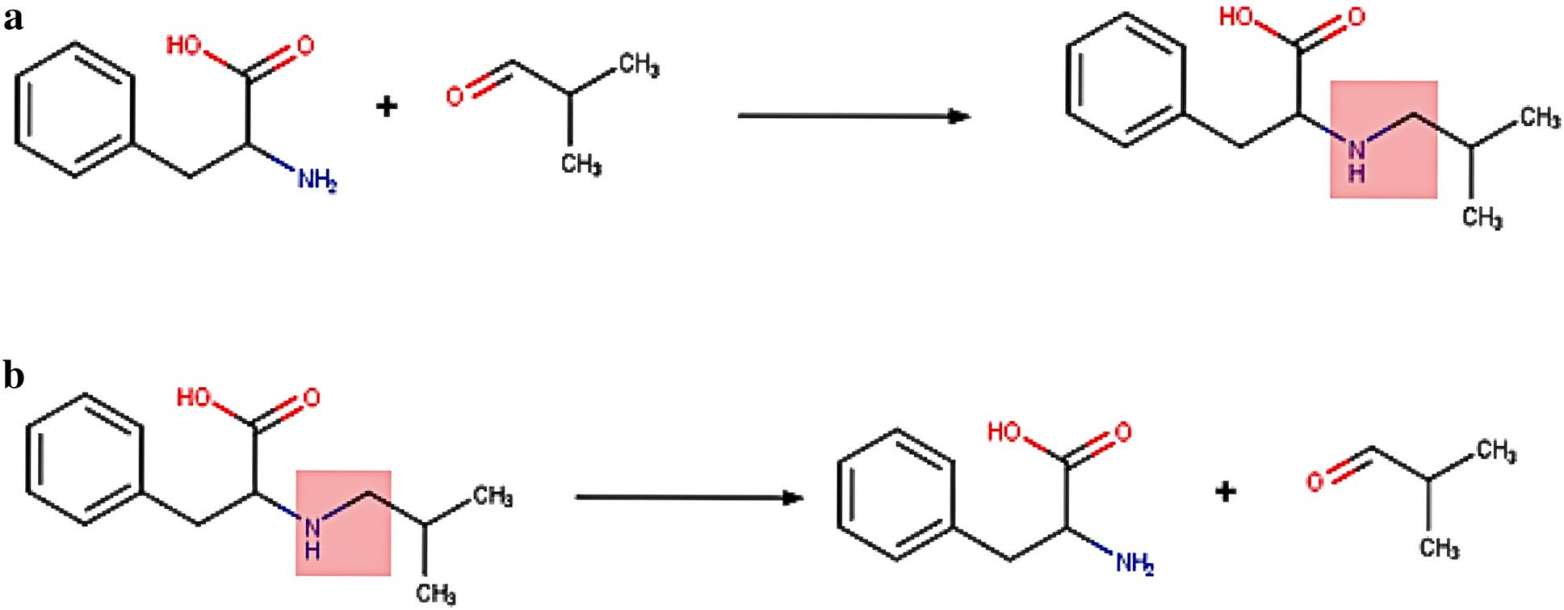
**a** A reaction example from US Patent 04703036 and **b** the reversed version of the same reaction. The reaction core is highlighted in red on the product side

The main aim of the training process is to produce Reverse Reaction Templates (RRT) for use during retrosynthetic analysis. As the name suggests, RRT’s are synthetic transformation rules which work conversely to decompose, i.e. transform, products into building blocks. Each RRT is derived from a class of highly similar reactions from which annotation data pertinent to the template, e.g. number of reactions, can be calculated. The process takes as input a set of reactions with atom mapping in place and, sequentially, reverses and standardizes the reactions followed by the extraction of an extended reaction core. During reversal, the two sides of the reaction, left-hand (reactants) and right hand (products) are exchanged. Agents, i.e. structures above and below the reaction line (e.g. catalysts, solvents), remain in their original place (Fig. 1).

**Fig. 2:**
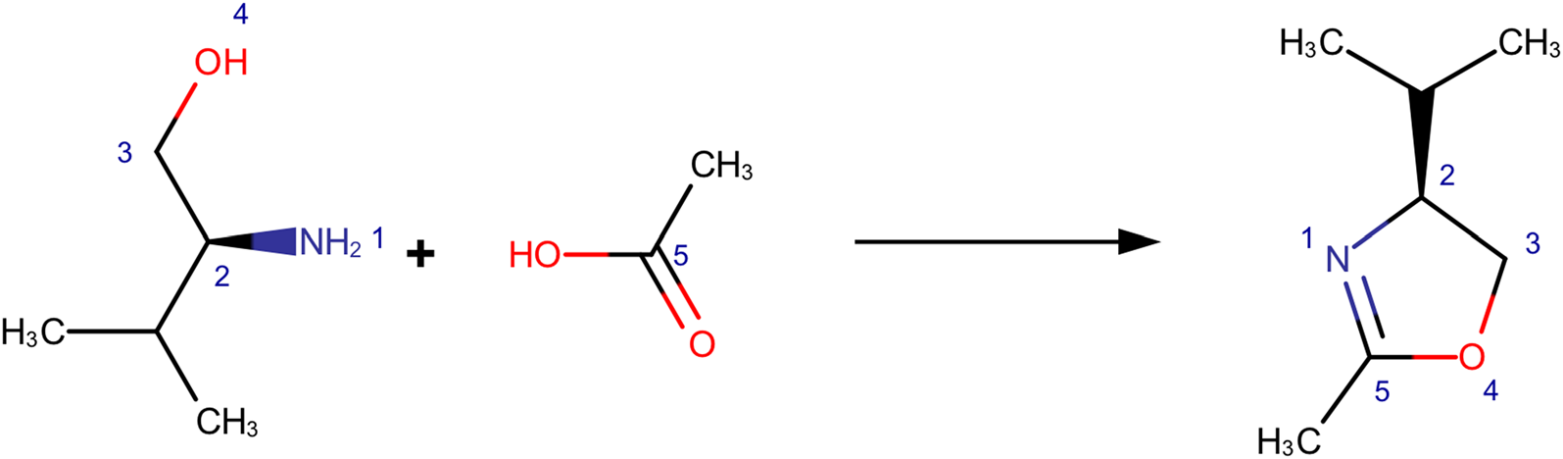
A heterocycle forming reaction from US patent number US20030149264A1. The reaction core consists of all numbered atoms 1, 2, 3, 4, 5

Standardization of the reaction includes, among others, removal of duplicate fragments, removal of fragments that do not participate in the reaction and handling reactions where there has been an obvious failure of the atom mapping process. The extraction of the extended reaction core requires that the main reaction core is first defined by identifying atoms whose properties (e.g. atomic symbol, connectivity, ring membership, etc) change during the reaction. If any of these atomic properties change from one side of a reaction that atom is considered a changing atom, part of the reaction core [8]. A reaction example illustrating the changing atoms forming the reaction core is shown in Fig. 2.

**Fig. 3:**
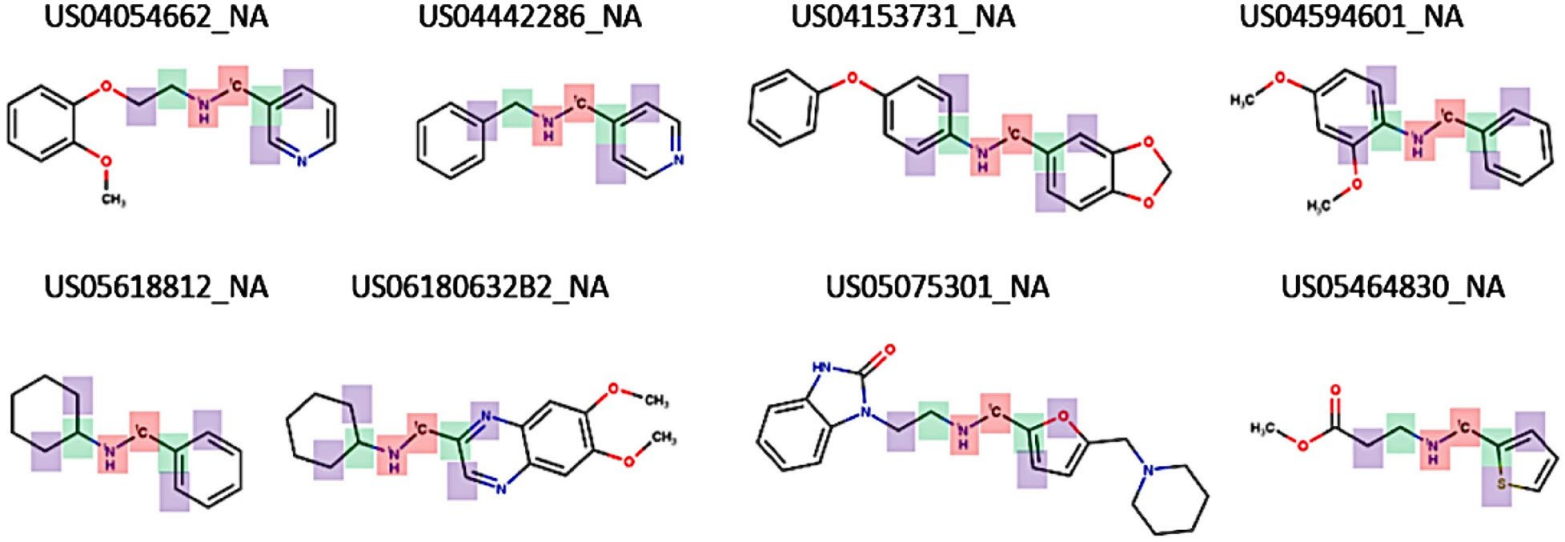
Reaction clusters defined using smiles-like signatures highlighted in red (radius 0), red and green (radius 1) and red, green and purple (radius 2). Note the effect of the atom properties used to the classification produced

In this case, all numbered atoms are considered changing atoms. Atoms 2 and 3 retain the same connectivity and neighbors during the reaction, but their ring membership changes, and given that ring membership is one of the atom properties perceived, those atoms are classified as changing, and part of the reaction core. If ring information were omitted from the atom typing used, they would not be perceived as part of the core. In order to accommodate varying user preferences the software has been implemented to allow flexible atom type definition. A full listing of the properties implemented can be found in Additional file 1: (SI 1).

A reaction is however not completely defined by just the changing atoms, or reaction core. Across a given reaction type, there will be a great diversity of different behaviors of such reactions such as varying yields, different catalysts needed, different solvents, temperatures, etc. These differences can be attributed to the larger context of the reaction core as embedded within the molecule. To capture more of this context, we apply a recursive Morgan-like expansion [11] around the reaction core, and capture reaction contexts of varying radii which, in essence, represent circular neighborhoods of different size. The radius zero context will be the reaction core itself, while a radius 3 context will include both the core atoms, and all atoms that are within 3 bonds of any atom in the core. The use of such shell-based reaction cores is common in retrosynthetic analysis applications as for example in [2] where they are referred to as reactive centers. In RTSA the extended reaction cores are represented using a unique smiles-like format referred to as signatures in this paper.

Reaction clusters are defined through grouping reactions sharing identical extended reaction cores at specific path lengths. Since extended reaction cores are represented as canonicalized smiles-like signature strings simple string matching suffices for the grouping operation. Clearly what gets grouped together will be a function of the radius selected. The larger the radius, the more “unique” reaction clusters there will be. Note that the use of a unique, canonicalized signature as the extended reaction core provides for a concise, easy to compare and comprehend representation that is flexible enough to accommodate any atom property needed. Figure 3 presents an example of reaction clusters defined using RTSA signatures.

Upon completion of this stage all input reactions have been reversed, standardized and their extended reaction cores have been extracted, represented as signatures and used to group reactions into homogeneous clusters. Each cluster, containing reactions sharing identical extended core is used to define retrosynthetic analysis transformation rules. Note that the choice of atom properties used to characterize each atom is crucial to the overall RTSA process since they influence the outcome of key steps such as the comparison of reaction extended cores and the definition of clusters (see “Results” section).

**Fig. 4:**
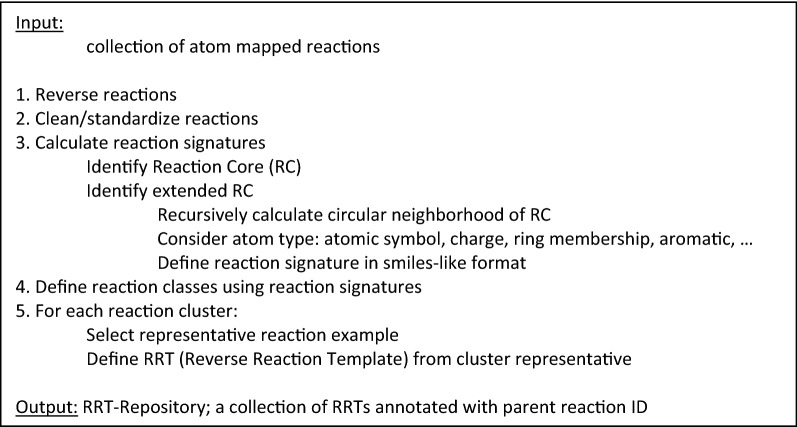
The RTSA-train process

Each reaction cluster is profiled using data from the reactions it contains. The resulting profile, typically summarization statistics on frequency and other available reaction properties, is stored for subsequent use as annotations to the corresponding reaction cluster. Finally, a single RRT template is selected from each cluster for use during retrosynthetic analysis. The choice of RRT from each cluster is insignificant as it is only used for retrieving the reaction signature that will serve as part of the retrosynthetic analysis transformation rule. The results of the training process are stored in an RRT-Repository for later use. Figure 4 outlines the steps of the RTSA-Train process. It is important to note that while the process may begin with very large numbers of reactions, this grouping procedure will typically result in far fewer representative reactions (see “Results” section).

### RTSA-design

**Fig. 5:**
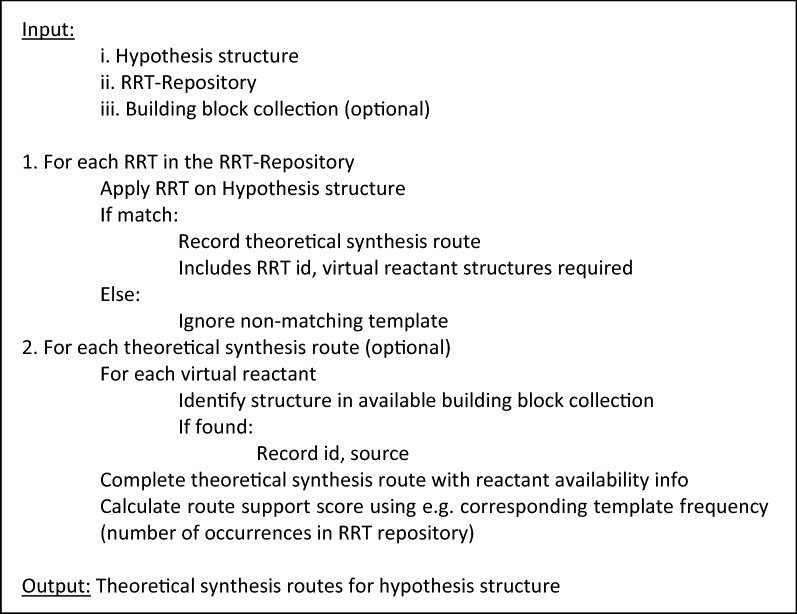
Pseudocode describing the high level RTSA-Design process. Note that step 2 is optional and could be omitted. Alternatively, the process may be supplemented with a recursive RTSA search for each of the synthons not found to be available leading to a multi-step synthesis route

During run time, the method takes an input hypothesis chemical structure and traverses the RRT-Repository attempting to run each reverse reaction template to decompose the structure. Attempts that result in failure due to no substructure match to the template, typically the overwhelming majority, are discarded. Successful RRT run attempts produce the structures of building blocks needed for the forward reaction to take place. In essence, each successful RRT run decomposes an input chemical structure into constituent reactants and provides information on the forward reaction type needed. Moreover, examples of past successful reactions of the same type are readily available through a lookup in the cluster of reactions of the matching RRT. In a subsequent step, the necessary building blocks derived from the application of an RRT may be searched for availability in accessible inventory databases. When available building blocks are found a synthetic route can be completed since necessary information required for attempting synthesis, including building blocks source and IDs, is available. Theoretical synthetic routes may be scored and ranked using annotation information (e.g. number of reaction examples, average yield) from the reaction cluster of the corresponding RRT. The RTSA-Design process is detailed in Fig. 5.

As described above the RTSA-Design process only searches for one-step synthetic routes since it requires that synthons resulting from successful RRT matching be present in accessible building block structure databases such as ZINC [7]. Alternatively, instead of aborting when building blocks are not available, the process may recursively treat such structures as new hypotheses to retrosynthetically analyze, thus enabling the process to identify multi-step synthesis routes. RTSA-Design could iterate until all synthons are found in a building block database, no successful RRT matching is found or some predefined depth is reached. As multi-step synthetic routes are more complex and thus more likely to fail in the lab they would typically be scored lower than single-step routes. However, such routes may serve as an idea generator to chemists searching for routes to novel chemical structures.

## Implementation

In this section we provide implementation details for key components of RTSA, namely *rxn_standardize* which cleans and standardizes reactions, *rxn_signature* which calculates reaction cores and *retrosynthesis* which retrosynthetically analyzes hypothesis structures using reverse reaction templates. The source code for these tools, as well as the procedure which reverses the reactions is provided through the LillyMol GitHub project at [4].

### rxn_standardize

Experience tells us that many sources of reactions contain significant amounts of unusable data, due to some combination of poor data entry, and/or errors in previous processing. The purpose of this tool is to ingest a stream of reactions, apply fixes where possible, or discard erroneous input. Since we are interested in retrosynthetic reactions, any forward reaction that produces multiple fragments as products will not be suitable and can be typically discarded. Also discarded are reactions where no structural change is observed between reactants and products, e.g. purifications. Where problems with the atom mapping are detected, for example duplicate atom map numbers, rxn_standardize provides an option to discard the existing atom mapping and, attempt to re-map the reaction. Although the built-in atom mapping was never intended to be comprehensive and is therefore not generally robust, this fix can “save” many reactions that would otherwise be discarded. Fragments not participating in the reaction, i.e. fragments with no atoms mapped or with no atoms whose type changes due to the reaction, can be removed. Similarly, atoms which either appear or disappear from the product side of the reaction can be handled so that subsequent RTSA tools can generate a correct and complete retrosynthetic path. Since the underlying LillyMol toolkit relies on Kekule representations of molecules, reactions can be switched to consistent Kekule forms in reagents and products. Duplicate fragments can also be discarded and other cleaning steps can be performed including removal of reactions with too large products or ensuring that the reaction name is a continuous alphanumeric string.

### rxn_signature

Extracting reaction signatures, the extended reaction cores, is a crucial step of the RTSA process. To achieve this the algorithm first assigns atom types to each atom. Atom type choice is user selectable, and the components of that type will determine whether or not an atom is considered a changing atom—part of the reaction core. The current implementation enables consideration of a large number of atomic properties to determine whether or not an atom has changed during a reaction including the atomic number, aromaticity, unsaturation, ring membership, formal charge, connectivity, smallest ring size, pi electron count and implicit hydrogen count (see Additional file 1 for a full listing of available atom properties). Once the reaction core atoms have been identified, the tool will record that set of atoms as the zero-radius reaction core. This is written as a form of unique smiles which contains the atomic symbol and a numeric label set by combining integer values corresponding to each of the contributing atomic properties (see “Results” section, Fig. 7). Subsequent layers are added to the core and those layers are also written, up to the maximum radius specified by the user. Since the tool writes a unique, text representation of the core, and variants, it is then straightforward to group reactions based on these representations via string matching methods.

After members of the same family of reaction are identified, a representative (template) at each radius can be selected. While the choice of template reaction is arbitrary, it will usually be desirable to select a reaction containing a relatively small number of atoms, just for clarity.

### retrosynthesis

Once a set of RRT reactions has been identified, based on the atom types and radius that has been selected, those template reactions can be used for deconstruction experiments. Template reactions are read in, changing atoms identified, and the signature, based on the specified radius, is converted to a substructure query representation. The defined substructure queries are subsequently used to process target molecules. Upon the presentation of a target molecule each reaction substructure query attempts a match. For those reactions which match, the corresponding retrosynthetic reaction is performed resulting in the deconstruction of the target molecule into one or more products. Those products, in essence the virtual reactant structures required to perform a reaction to generate the target molecule, can then be looked up in any available structure database.

Note that a number of the steps in the process can be run using parallel processing techniques to reduce required run time. For example, each reaction template can be matched independent of the other reactions.

## Results

In order to present the capabilities of RTSA we have selected to train the method on a set of publicly available reactions and provide application examples illustrating the results it can produce. The current source code implementation of RTSA is available [4] including useful wrappers for the analysis presented below. All times reported below were obtained on runs performed on a Linux workstation with an Intel Xeon 12-core processor and 64 GB RAM.

### Data

Dataset 1, a set of 1,808,938 publicly available reactions, has been used for training RTSA. The set consists of reactions in patent grants from 1976–2016 available at [10]. Dataset 1 was prepared through text mining reactions from patents of the United States Patent and Trade Office followed by structure processing and atom mapping as described by Lowe [10]. Dataset 2 consists of 919 approved drugs from DrugBank 4.3 Wishart et al. [20] satisfying the Lilly medicinal chemistry rules [1], stripped of salts and filtered to structures with a range of heavy atoms between 20 and 40. Dataset 2 was used to test the retrosynthetic analysis calculation of RTSA as trained using Dataset 1. Note that 492 of the 919 (53.5%) structures in Dataset 2 are also present as products in Dataset 1 reactions.

### RTSA-train on patent reactions

In a first step, Dataset 1 was processed to generate a file containing reaction smiles followed by a unique ID. The patent reactions, already atom-mapped as described in [10], were then standardized using rxn_standardize. The reactions were then reversed, transformed to contain “+” instead of “.” symbols as required by the RTSA code and subjected to a second standardization process, also with rxn_standardize. A total of 293,008 reactions were rejected during standardization for reasons mostly related to the presence of multiple products or for having a too large product (our standardization only allowed products with a maximum size of 40 heavy atoms). Reaction signatures were calculated for the remaining 1,515,930 successfully reversed and standardized reactions using the rxn_signature tool for radii 0, 1 and 2. The atom types chosen captured information for atomic number (Z), aromaticity (A), number of connections (C), formal charge (O), ring bond count (R), smallest ring size (S) and unsaturation (U) which is the current default setting of this tool. Reaction signatures were successfully generated for all of the attempted reactions. In order to obtain reverse reaction templates simple string matching was used to compare reaction signatures at each radius and define unique instances and their frequency count. Through this process a total of 83,942 unique signatures were defined for radius 0, 180,862 for radius 1 and 325,873 for radius 2. Each unique reaction signature represented a group of reactions with size 1 or greater.

**Fig. 6:**
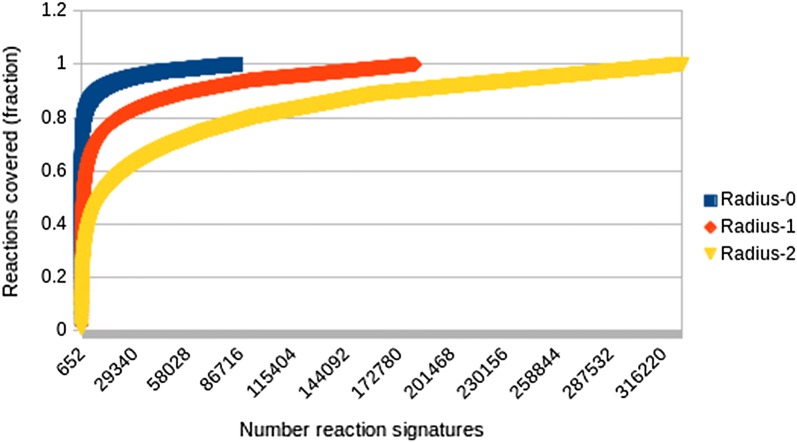
Fraction of reaction examples covered as a function of number of reaction signatures for different radii sizes. Note the steep curves indicating that a large fraction of the reactions examined is covered by relatively few reaction signatures

The coverage of each RRT set, i.e. the number of reactions whose signature is represented in the RRT set, elsewhere referred to as condensation numbers [2], provide insights for the diversity of the reaction types in Dataset 1. Figure 6 plots the fraction of reaction examples covered by reaction signatures of different size. As shown a small number of reaction signatures cover a large number of reaction examples with less specific signature types such as those at radius 0 exhibiting a higher coverage as expected. This finding is in agreement with results reported previously by Law et al. [8] and Christ et al. [2] among others.

In order to increase our confidence in the reaction transformation rules represented by the various signatures we post-processed the results to select subsets which have varying numbers of supporting examples. Table 1 displays the number of reaction signatures (and RRT) for Dataset 1 reactions for radii 0, 1 and 2 with support of 10, 100, 1000 and 10,000 examples. The subset of reaction signatures of radius 0 supported by at least 10 examples is just 7945 out of 83,942 (9.4%). The corresponding numbers for radii 1 and 2 are 14,567 (8%) and 20,499 (6.3%). This very large compression from the initial starting set goes a long way towards improving run time performance during RTSA-Design and addressing the scalability issue faced by direct search methods. Moreover, high support of a template indicates popularity of the corresponding reaction among practicing chemists and, thus, increased confidence in the synthetic routes proposed by the subsequent RTSA-Design step. The python script used for preparation of RRT repositories from patent grants reactions can be found in SI 2  (see Additional file 2).

**Table 1:** RRT sets extracted used for RTSA validation

Template set	Radius	Support (minimum number of reaction examples per template)	Size
RRT-0-1	0	1	83,942
RRT-0-10	0	10	7945
RRT-0-100	0	100	960
RRT-0-1000	0	1000	165
RRT-0-10000	0	10,000	21
RRT-1-1	1	1	180,862
RRT-1-10	1	10	14,567
RRT-1-100	1	100	1614
RRT-1-1000	1	1000	144
RRT-1-10,000	1	10,000	4
RRT-2-1	2	1	325,873
RRT-2-10	2	10	20,499
RRT-2-100	2	100	1264
RRT-2-1000	2	1000	57
RRT-2-10,000	2	10,000	3

**Fig. 7:**
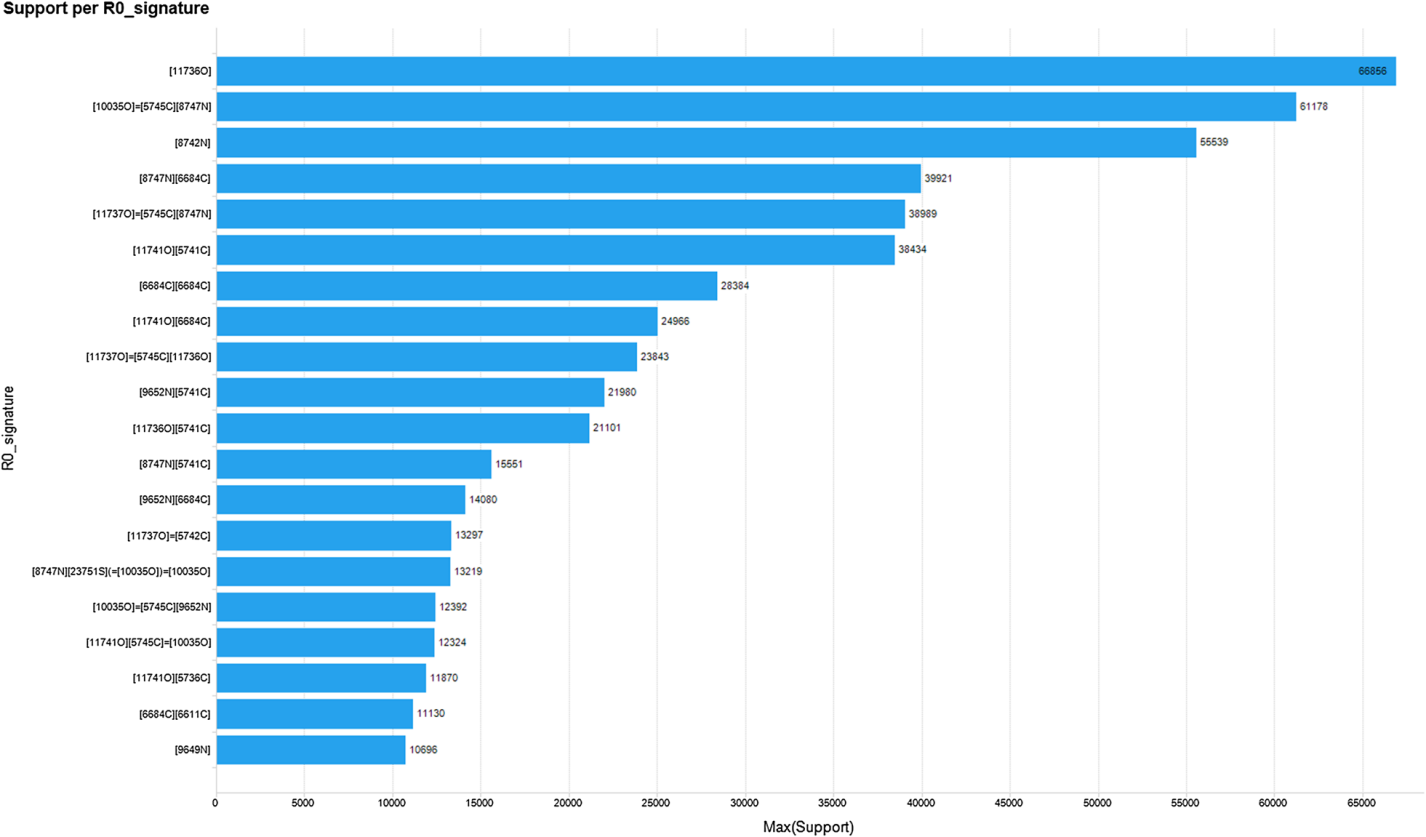
Example RRT’s extracted using signatures of radius 0 and support 10,000

**Fig. 8:**
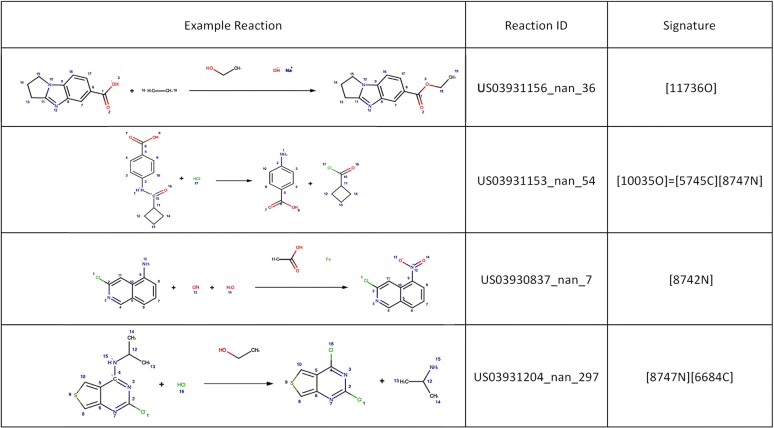
Examples of reverse reaction templates exemplifying the 4 signatures of radius 0 with the highest support in Dataset 1. Note that the templates are reversed, i.e. the original products are to the left-hand side of the figure

Visual inspection of the reaction templates reveals that common reaction types are captured by the RRT’s extracted. Figure 7 presents the reaction signatures corresponding to the RRT of radius 0 with the highest support, i.e. frequency of reaction examples. Examples of reverse reaction templates sharing the 4 signatures with the highest support are shown in Fig. 8. As shown, the most supported reaction type involves a transformation rule with a single changing Oxygen atom, in this case an ester hydrolysis (Fig. 8a). Next is the amide coupling signature exemplified by a Schotten–Baumann reaction for the synthesis of an amide from amine and acid chloride (Fig. 8b), a reaction with a single changing nitrogen shown by a nitro reduction (Fig. 8c) and a nucleophilic aromatic substitution widely known as SnAR (Fig. 8d).

### RTSA-design application on approved drugs

In order to assess the RTSA methodology capabilities Dataset 2 containing 919 FDA approved drugs has been subjected to retrosynthetic analysis using the RRT-Repositories prepared by training on Dataset 1 reactions and the tool RTSA (see Additional file 3: SI 3). The purpose of RTSA is to support synthetic chemists in identifying realistic routes to prepare a compound of interest. With this purpose in mind, we chose Dataset 1, a typical set of compounds, as targets and assess the success of RTSA both quantitatively, i.e. number of routes found per target and qualitatively, via reviewing the specific routes of a compound of interest.

Overall, RTSA-Design successfully identified theoretical synthesis routes for the majority of the query structures. This comes as no surprise since the reaction collection used for RRT extraction is both diverse and substantially extensive as shown in the training section above. Note that for the purposes of this RTSA-Design demonstration no synthon look-up in building block databases was undertaken so all theoretical routes identified are reported. In a realistic scenario, where actual synthesis might have been considered, a search for the identification of synthons in available structure collections would have indicated which of the theoretical routes could potentially be executed. Detailed results of the retrosynthetic analysis runs are displayed in Table 2 below.

**Table 2:** Synthetic route identification for 919 known drug structures using RTSA-Design and a range of RRT-Repositories

RRT set	RTSA results (structures with at least one route)	Coverage	Average number of routes
RRT-0-10	917	917/919	922,449/917
RRT-0-100	917	917/919	101,309/917
RRT-0-1000	917	917/919	21,255/917
RRT-0-10000	831	831/919	3319/831
RRT-1-10	916	916/919	24,209/916
RRT-1-100	905	905/919	9497/905
RRT-1-1000	835	835/919	4076/835
RRT-1-10000	327	327/919	423/327
RRT-2-10	841	841/919	6620/841
RRT-2-100	756	756/919	2344/756
RRT-2-1000	284	284/919	455/284
RRT-2-10000	52	52/919	53/52

As expected the tool successfully decomposes more structures from Dataset 2 using templates extracted with radius 0 which are more abstract than the more specific templates of radius 1 and 2. Similarly, the subsets of templates with higher support have a smaller coverage when compared to template subsets with less support since the former are fewer in absolute numbers. However, the level of confidence in the proposed routes is higher for those proposed by the most specific RRT, in this case those of radius 2 with higher support. In the case of the RRT set with support of at least 1000 reactions RRT’s of radius 0 have a near 100% coverage (917/919) while RRT’s of radii 1 and 2 identified routes for 835 and 284 of the drug structures which corresponds to 90% and 31% respectively. A similar trend of decreasing coverage when using templates of increasing support is observed for all radii. Overall, compromising with a minimum support of 100 reaction examples exhibited adequate coverage even for radius 2 (756/919; 82%). Time requirements for RTSA-Design of Dataset 2 varied depending on the RRT set used. As expected the least time, less than 2 s, was required for running the RRT-2-10,000 set with only 3 templates that resulted in 53 route definitions. The use of RRT-0-10 with 7945 templates that resulted in 922,449 routes took approximately 6 min while set RRT-2-10 with 20,499 templates producing 6620 routes required only 75 s. It can thus be concluded that the time needed for RTSA-Design runs is proportional to the number of routes identified and that higher numbers of RRT do not necessarily translate to longer run times especially if those templates are highly specific as in the case of using signatures of radius 2 or higher. In general, templates representing smaller, more abstract signatures will match more frequently to hypothesis chemical structures, trigger the generation of more theoretical routes and require more time. This observation can also be seen in Table 2 listing the average number of routes theoretically possible per input target structure which corresponds to the template matches to each target structure in the test set. As noted previously the training set contains reactions for little over 50% of the compounds in the test set. Analysis of the RTSA-Design results showed that the method was able to identify the original routes for those compounds (subject to signature frequency restrictions) and, additionally, provide alternative synthetic paths.

**Fig. 9:**
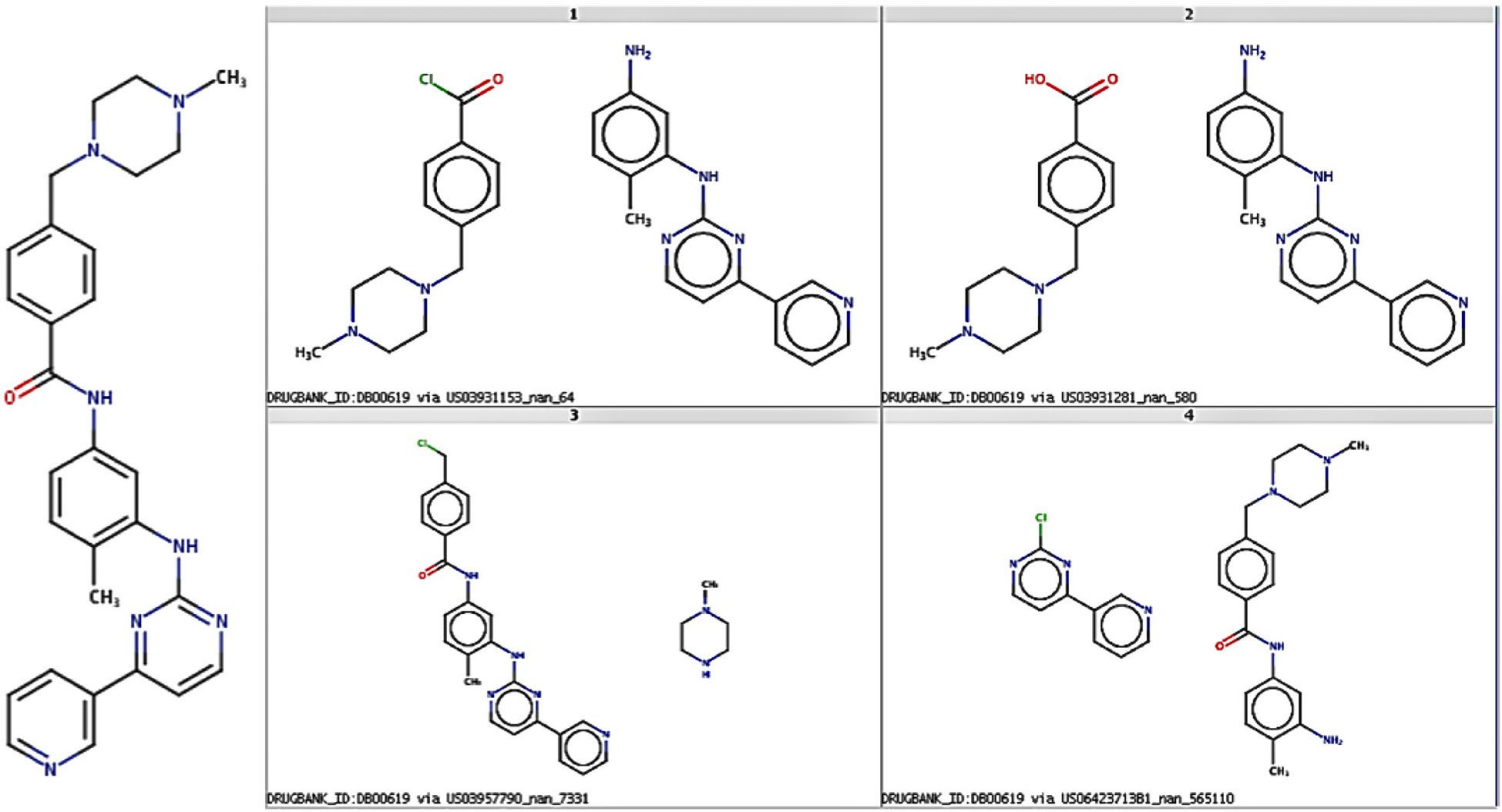
DrugBankID:DB00619 and four potential synthesis routes identified by RTSA using reverse reaction templates of signature radius 2 and frequency 100 extracted from Dataset 1. Note that the origin of the reverse reaction template used to define the route is shown below each pair of synthons

Figure 9 below presents multiple theoretical synthetic routes using radius 2 and support 100 for Gleevec (DrugBankID:DB00619), one of the 919 drugs in the test set used in this exercise. The four routes shown, a selection of those proposed, present the variety of synthetic routes possible.

The four routes shown in Fig. 9 represent common reaction types employed by medicinal chemists. Each of the routes is displayed as a pair of synthons which, when processed with appropriate conditions will react to form the target compound shown to the left. The first of the routes, top-left, corresponds to an amide Schotten–Baumann synthesis combining an acid chloride and a primary amine. The second route shown at top-right, is another common amide bond formation via the combination a primary amine with a carboxylic acid. Route 3, bottom-left, uses an amine and an alkyl halide in a reaction widely known as N-alkylation. The final route shown, bottom-right, is a nucleophilic aromatic substitution (SnAR) combining a primary amine with a NAS-electrophile. A look-up of the synthons into the popular ZINC database indicated that all of the reactants are available for purchase (see Additional file 4: SI 4).

The source of the reaction transformation rule, i.e. the original reaction example that was used to define the corresponding RRT, is shown in the description accompanying each of the synthon pairs. For example, the source for the top-left route is US03931153_nan_64 (USPTO patent number US03931153, paragraph number not reported—‘nan’, row number in input reaction examples file 64).

## Conclusions

The need for synthetic route identification arises frequently in drug discovery and development as well as other chemical processes involving new chemical structure design or identification of optimal synthetic paths. For inspiration, chemists traditionally have resorted to product or reaction-based search in collections of past reaction examples. Recently, several computational methods capable of designing synthetic routes have been reported in the literature [2, 3, 8, 18, 19]. In this paper we have presented RTSA, a data-driven RA technique developed to address the needs of a fast-paced discovery chemistry organization. RTSA learns RRT’s from collections of reaction examples and applies them for the deconstruction of target structures into synthons. Post-processing of the identified routes, for example searching for the presence of synthons in available building block databases or using any of the synthons as input to a new RTSA search can optionally be applied depending on the specific use case.

The implementation of RTSA as well as a fully functional example of its application to learn from a public patent reaction collection and to identify possible routes for a set of known drugs is provided as part of this paper. As demonstrated in this example RTSA can quickly derive RRT’s of varying length and complexity from millions of reaction examples and identify routes for large numbers of target compounds in minutes. The speed, flexibility and reaction rule interpretation simplicity of RTSA enable its regular use during structure design so as to inform research chemists of the feasibility of their designs and, thereby, the ease of experimental testing of their hypotheses.

It is the opinion of the authors that the impact of data-driven computational approaches in synthetic route design and drug discovery research at large will only increase in the coming years. Software tools that can learn from prior synthetic attempts and apply these learnings to identify feasible synthetic paths, such as RTSA, will routinely support researchers in efforts to synthesize novel chemical structures or identify alternative viable routes to a compound of interest. Combined with advances in automated synthesis systems [6] and algorithmic methods for in silico drug design [12] RTSA-like methods have the potential of revolutionizing everyday discovery and development practices in the not so distant future [13].

## Additional files

**Additional file 1.** Supporting information 1 (SI1) contains a list of all the atomic properties supported by RTSA to determine changing atoms during a reaction; used for reverse reaction template definition and application.

**Additional file 2.** SI2 contains the python script rtsa_train.py which was used for the preparation of RRT repositories as described in the Results section of this paper.

**Additional file 3.** SI3 lists the commands used to retrosynthetically analyze the test set of approved drugs as described in the Results section of this paper.

**Additional file 4.** SI4 includes the structure and ZINC numbers for all building blocks shown in Fig. 9.

## Abbreviations

RA: Retrosynthetic analysis
eLN: electronic laboratory notebook
RTSA: ReTroSynthetic analysis method
RRT: Reverse reaction template
