# Supplementary material for: A retrosynthetic analysis algorithm implementation
Source: J Cheminform. 2019 Jan 3;11:1. doi: 10.1186/s13321-018-0323-6 (PMC6689887; doi:10.1186/s13321-018-0323-6)
Supplement: Supplementary file 1 — Additional file 1. Supporting information 1 (SI1) contains a list of all the atomic properties supported by RTSA to determine changing atoms during a reaction; used for reverse reaction template definition and application. [file 13321_2018_323_MOESM1_ESM.docx]

Supporting Information 1: List of all the atomic properties supported by RTSA to determine changing atoms during a reaction; used for reverse reaction template definition and application.

A aromatic or not

C number of connections

E carbon atoms get one type, all heteroatoms a different type

F ring fusion

G atomic symbol hash

H implicit hydrogen count

I isotope

K atomic numbers of connected atoms

L largest ring

M all aromatic atoms the same

N all atoms get the same type

O formal charge

P pi electron count

Q presence or absence of a pi electron

R ring bond count

S smallest ring

T atomic number, but possibly tautomeric nitrogens grouped

U unsaturation

X centrality of atom (expensive, uses distance matrix)

Y atomic number, heavy halogens compressed

Z atomic numbers
