# Supplementary material for: A retrosynthetic analysis algorithm implementation
Source: J Cheminform. 2019 Jan 3;11:1. doi: 10.1186/s13321-018-0323-6 (PMC6689887; doi:10.1186/s13321-018-0323-6)
Supplement: Supplementary file 2 — Additional file 2. SI2 contains the python script rtsa_train.py which was used for the preparation of RRT repositories as described in the Results section of this paper. [file 13321_2018_323_MOESM2_ESM.docx]

Supporting Information 2: python script rtsa_train.py used for preparation of RRT repositories from patent grants reactions (Dataset 1) as described in the Results section of paper.

#!/usr/bin/env python

########################################################################

# Copyright 2018 Eli Lilly and Company

#

#

# Licensed under the Apache License, Version 2.0 (the "License");

# you may not use this file except in compliance with the License.

# You may obtain a copy of the License at

#

# http://www.apache.org/licenses/LICENSE-2.0

#

# Unless required by applicable law or agreed to in writing, software

# distributed under the License is distributed on an "AS IS" BASIS,

# WITHOUT WARRANTIES OR CONDITIONS OF ANY KIND, either express or implied.

# See the License for the specific language governing permissions and

# limitations under the License.

########################################################################

# Version date: 20180822

import numpy

import argparse, subprocess, os, string

import pandas as pd

def main():

"""Module mainline (for standalone execution)"""

parser = argparse.ArgumentParser()

parser.add_argument("-o", "--productFileName", help="file containing RRT", required=True )

parser.add_argument("-i", "--inputFile", help="input reactions file", default=None)

parser.add_argument("-r", "--radius", help="reaction core/signature radius", type=int, choices=[0,1,2], default=0)

parser.add_argument("-t", "--minThreshold", help="minimum size of signature group, support", type=int, default=2)

group = parser.add_mutually_exclusive_group()

group.add_argument("-sr", "--signatureReady",

help="input file in format ready for signature extraction (cleaned, reversed, plus instead of dot, standardized)",

action="store_true", default=False)

group.add_argument("-tr", "--templateReady",

help="input file in signature format ready for RRT template extraction",

action="store_true", default=False)

parser.add_argument("-v", "--verbose", help="run in debug, verbose mode", action="store_true", default=0)

parser.add_argument("-d", "--debug", help="enable debug mode", action="store_true", default=0)

# get the arguments

args = parser.parse_args()

if args.verbose:

print "Arguments supplied...", args

# A. Prep local environment, initial rsmi file from patents

# local vars

outpathstem = os.path.split(args.productFileName)[0]

outpathfn = os.path.split(args.productFileName)[1]

outpathbase = os.path.splitext(args.productFileName)[0]

if args.verbose:

print "Outgoing filenames/stems...", outpathstem, outpathfn, outpathbase

# preparing names

out_clean = outpathbase + '_clean_1.rsmi'

out_fstd = os.path.join(outpathbase + '_fstd_2.rsmi')

out_lrsmi = os.path.join(outpathbase + '_plusfstd_3.rsmi')

out_rev = os.path.join(outpathbase + '_rev_4.rsmi')

out_rstd = os.path.join(outpathbase + '_rstd_5.rsmi')

out_sig = os.path.join(outpathbase + '_rstd_sig_6.txt')

out_sig_counts = os.path.join(outpathbase + '_rstd_sig_counts_6.txt')

out_sig_bad = os.path.join(outpathbase + '_rstd_sig_bad_6.txt')

# raw reaction file supplied; preprocess, extract signatures, extract templates

if args.templateReady is False and args.signatureReady is False:

preprocess(args.inputFile, out_clean, out_fstd, out_lrsmi, out_rev, out_rstd, args.verbose)

extractsignatures(out_rstd, out_sig, out_sig_counts, out_sig_bad, args.verbose)

# reactions are preprocessed only proceed to extract signatures and templates

elif args.signatureReady and args.signatureReady is False:

# preparing names

sroutpathbase = args.inputFile

out_rstd = os.path.join(sroutpathbase + '_rstd_5.rsmi')

extractsignatures(out_rstd, out_sig, out_sig_counts, out_sig_bad)

# reaction are preprocessed and signatures have been extracted; proceed to template extraction

elif args.templateReady:

# preparing names

sroutpathbase = args.inputFile

#print '>>>', sroutpathbase

out_rstd = os.path.join(sroutpathbase + '_rstd_5.rsmi')

out_sig = os.path.join(sroutpathbase + '_rstd_sig_6.txt')

## RRT extraction - threshold-based

## (supplying signature file, std reaction for using in the final RRT file, the output RRT file, ...)

pickpopulous(out_sig, out_rstd, args.productFileName,

radius=args.radius, min_threshold=args.minThreshold, verbose=args.verbose)

return

#

# given a raw rsmi file

# - clean

# - convert dots to pluses

# - reverse from reactants>agents>products to products>agents>reactants

# - standardize

#

def preprocess(infile, out_clean, out_fstd, out_lrsmi, out_rev, out_rstd, verbose=0):

rxdf = pd.read_csv(infile, sep='\t', header=None, error_bad_lines=False, warn_bad_lines=False)

# cleaning additional symbols from rsmiles column

rxdf[[0,'Extensions']] = rxdf[0].str.split(' ', expand=True)

# generating new, unique id column

rxdf['RowNum'] = range(0, 0 + len(rxdf))

rxdf['id'] = rxdf[1].astype(str) + '_' + rxdf[2].astype(str) + '_' + rxdf['RowNum'].astype(str)

# replacing . with +

rxdf.to_csv(out_clean, sep=' ', index=False, header=False, columns=[0,'id'])

# B. RTSA-Train

## standardize forward reactions; remove salts, fragments, make sure fully mapped (20180822), ...

if verbose:

print "\nrxn_standardize forward reactions..."

cmd = "rxn_standardize.sh -v -c -X igbad -X fmap -s -K " + out_clean + " > " + out_fstd + " 2>" + out_fstd + ".log\n"

if verbose:

print cmd

subprocess.call([cmd], shell=True)

## convert to lrsmi format;

if verbose:

print "\nConverting to plusrsmi representation..."

cmd = 'sed "s/\./+/g" ' + out_fstd + ' > ' + out_lrsmi

if verbose:

print cmd

subprocess.call([cmd], shell=True)

## reverse: rsmi to revrsmi

ins = open(out_lrsmi)

outs=open(out_rev,'w')

for line in ins:

outline = reverse_smi(line)

outs.write(outline)

outs.close()

## standardize reversed reactions

if verbose:

print "\nrxn_standardize reverse reactions..."

cmd = "rxn_standardize.sh -s -D x -X igbad -X nclf -v -C 40 -K -E autocreate -e -o -m -I -b " \

+ out_rev + " > " + out_rstd + " 2>" + out_rstd + ".log\n" # -X rmdup -D u

if verbose:

print cmd

subprocess.call([cmd], shell=True)

return

#

# given an rsmi or lrsmi (reaction smiles string)

# reverse the parts and return the result

#

def reverse_smi(rsmi, debug=False):

# separate into reaction and name

rxnAndName = rsmi.split(" ")

if len(rxnAndName) < 2:

raise ValueError("Error in smiles - Expected a smiles string followed by a space , then a compound name")

thisRxn = rxnAndName[0]

# now split the rxn parts - reactants, agents, and products

rxnParts = thisRxn.split(">") # split the rxn part

if len(rxnParts) != 3:

raise ValueError('Error in reaction smiles -expected three parts : "' + rsmi + '"')

outString = rxnParts[2] + '>' + rxnParts[1] + '>' + rxnParts[0]

for nameIndex in range(1, len(rxnAndName)):

# name might have had spaces and so would be split into multiple parts

outString += ' ' + rxnAndName[nameIndex]

return outString

#

# given clean, reversed, standardized, with plus instead of dots rsmi file

# extract reaction signatures

#

def extractsignatures(out_rstd, out_sig, out_sig_counts, out_sig_bad, verbose=0):

if verbose:

print "\nrxn_signature..."

cmd = "rxn_signature.sh -v -r 0,1,2 -C " + out_sig_counts + \

" -F " + out_sig_bad + " " + out_rstd + " > " + out_sig + " 2> " + out_sig + ".log"

if verbose:

print cmd

subprocess.call([cmd], shell=True)

return

#

# pick only signatures with at least <min_threshold> instances

#

def pickpopulous(sigfile, rsmistdfile, outfile, radius=0, min_threshold=2, verbose=0):

radius = 'sigr'+str(radius)

# read signature file

cdf = pd.read_table(sigfile, delim_whitespace=True, header=None)

if verbose:

print "\ncdf:", cdf.shape, cdf.columns

cdf.columns = ['sigrsmi', 'NA', 'id', 'sigr0', 'sigr1', 'sigr2']

if verbose:

print "\ncdf:", cdf.shape, cdf.columns

# get signature groups/distribution

try:

groups = cdf.groupby(radius).size()

except:

print "Radius problem; rad", radius, "column does not exist! Aborting ..."

# find signatures meeting frequency threshold and

# write frequency distribution for each signature string in file for reference

out_distr = outfile + ".freqdistr_7"

outs = open(out_distr, 'w')

sig_list = []

for sig, count in groups.iteritems():

outs.write(sig + ' ' + str(count) + '\n')

if count >= min_threshold:

#print sig, ":", count

sig_list.append(sig)

outs.close()

if verbose:

print "Number of signatures meeting threshold", min_threshold, "is", len(sig_list)

# get the new dataframe with first of each signature group # this is not a normal dataframe!!!!!!

scdf = cdf.groupby(radius, as_index=False).first()

if verbose:

print "\nscdf:", scdf.shape, scdf.columns #, scdf.iloc(0).to_string()

# prep sub-dataframe with RRT corresponding to signatures meeting threshold; 0 column look-up since we reset index above

fscdf = scdf.loc[scdf[radius].isin(sig_list)]

if verbose:

print "\nfscdf:", fscdf.shape, fscdf.columns #, fscdf.iloc(0).to_string()

# load dataframe to pull rsmi from

stddf = pd.read_table(rsmistdfile, delim_whitespace=True, header=None)

stddf.columns = ['stdrsmi', 'id']

# merge frequency filtered sub-dataframe with dataframe containing proper rsmi

mergedf = pd.merge(fscdf, stddf, on='id', how='left')

if verbose:

print "\nmergedf:", mergedf.shape, mergedf.columns

# write to RRT file; 0 column is reaction template, 2 is the id

mergedf.to_csv(outfile, sep=' ', index=False, header=False, columns=['stdrsmi','id']) #, 'sigrsmi','radius'])

# record for reference

fscdf.to_csv(outfile+"_fscdf_7", sep=' ', index=False, header=False)

mergedf.to_csv(outfile+"_mergedf_7", sep=' ', index=False, header=False)

return

#

#

#

if __name__=="__main__":

main()
