# Supplementary material for: A retrosynthetic analysis algorithm implementation
Source: J Cheminform. 2019 Jan 3;11:1. doi: 10.1186/s13321-018-0323-6 (PMC6689887; doi:10.1186/s13321-018-0323-6)
Supplement: Supplementary file 3 — Additional file 3. SI3 lists the commands used to retrosynthetically analyze the test set of approved drugs as described in the Results section of this paper. [file 13321_2018_323_MOESM3_ESM.docx]

Supporting Information 3: Commands used to retrosynthetically analyze the test set of approved drugs (Dataset 2) as described in the Results section of this paper.

Tool retrosynthesis.sh was use to retrosynthetically analyze the drug compounds in Dataset 2. The general structure of the command used is:

retrosynthesis.sh -Z -z -Y all -X rmiso -X kg -X kekule -X ersfrm -a 2 -q f -R <R> -I 1976_Sep2016_USPTOgrants_smiles_t<T>_r<R>.rsmi -P UST:AZUCORS 20150827_approved.smi > approved_t<T>_r<R>.rsmi 2>approved_t<T>_r<R>.log

where:

<R> should be replaced by radius size, i.e. 0, 1 or 2

<T> indicates the level of support, i.e. 10, 100, 1000, 10000

-Z ignore reactions that cannot be constructed

-z ignore reactions with no changing atoms

-Y all apply all available chemical standardization options to output

-X rmiso remove isotopes from incoming molecules

-X kg keep going after a test failure

-X kekule preserve Kekule forms

-X ersfrm add an extra line in output with small fragments removed

-a 2 do NOT write products with fewer than 2 atoms

-q f use the reaction file name as the reaction name

-R <R> radius used for reaction signature definition

-I file of Reverse Reaction Templates

-P UST:AZUCORS atomic properties to determine changing atoms
