# Supplementary material for: A retrosynthetic analysis algorithm implementation
Source: J Cheminform. 2019 Jan 3;11:1. doi: 10.1186/s13321-018-0323-6 (PMC6689887; doi:10.1186/s13321-018-0323-6)
Supplement: Supplementary file 4 — Additional file 4. SI4 includes the structure and ZINC numbers for all building blocks shown in Fig. 9. [file 13321_2018_323_MOESM4_ESM.docx]

Supporting Information 4: ZINC numbers for building blocks of the Gleevec synthesis routes shown in Figure 9.

The ZINC numbers were obtained by simple look-up of the SMILES structures produced by RTSA-Design for the four Gleevec routes shown in Figure 9. All building blocks were reported available for purchase on November 12, 2018.
